# A Case of Anasarca and Hydrops Pulmonum

**Published:** 1802-05

**Authors:** William Coley

**Affiliations:** Surgeon, in Bridgnorth


### *A Case of Anasarca and Hydrops Pulmonum,*

*Which were principally removed by Scarifications; but were, after some months, succeeded by an Ascites and Hydrops Vaginæ, which latter form of the disease was cured by tapping the Vagina. In two parts; with introductory observations to each. Communicated by* Mr. WILLIAM COLEY, *Surgeon, in Bridgnorth.*

INTRODUCTORY to the following hiftory, I beg leave to make fome obfervations on the practice of *scarifications* in hydropic difeafes; of which this cafe bears a moft convincing teftimony of their wonderful utility, even in a fituation the moft defperate and unpromifing. This is the more neceffary, as from my own knowledge I find that the provincial practitioners, in general, are extremely averfe to the practice, notwithftanding fo much was formerly advanced in its favour by the late Dr. Fothergill. *(Med. Obs. & Inq. vol. iv.)* So far as the evidence of an individual can go, whofe practice is regulated, not fo much by theory, or theorizing, as by a clofe and impartial obfervance of the effects and operations of remedies in difeafes, I can truly affirm that in no inftance, of more than fifty anafarcous cafes in which I have perfonally feen and ufed the fcarifications, or incifions, have I in any one ever obferved the leaft injury to arife from them, though they have at one time or other been employed upon almoft every external part of the human body; they have fometimes been reforted to at too late a period of difeafe, and what remedy occafionally has not? but in almoft every fair cafe that has occurred, that is, in every fituation where there could be a poffibility of relief expected, much good has been done by them; many cafes of anafarca of the thighs and legs have been cured chiefly by this remedy; and œdematous fwellings, in any fituation of the external parts, have generally been alone removed by them. The marks occafioned by the incifions after they are healed become fcarcely vifible, though the operation may have been many times repeated. The operation is moft conveniently done, where the part will admit of its application, by the common fcarificator; but where it will not, fmall incifions, not exceeding one-fourth of of an inch in length, made juſt through the ſkin with the point of a lancet, do equally well. They ſhould be made not in the direction of the muſcular fibres, but tranſverſely. Prepared cabbage leaves, or ſimple cerate, ſhould be kept over the inciſions to preſerve the orifices moiſt, and thus prevent their prematurely cloſing. The inciſions commonly keep open from two to four or five days; and it is wonderful to obſerve in many caſes what a ſurpriſing quantity of liquid diſcharges from them. I cannot therefore too ſtrongly recommend, in addition to the high authority before mentioned, an extenſion of the practice of ſcarifications in all ſimilar caſes.

*The First Part of the Case, or that which relates to the Anasarca and the Hydrops Pulmonum only.*

1800, April 9, Mrs. P. a woman of a very debile and delicate habit, moderate ſtature, thin ſkin, grey eyes; mother of two very weakly living children; had been ſubject to repeated abortions. She lay-in about five weeks ago, previous to which there was a conſiderable œdema of the legs. She has now an almoſt univerſal dropſy, which has been gradually increaſing ever ſince her delivery. The thighs, legs and feet, are ſwollen to a moſt enormous ſize, and are nearly cold; but though ſo greatly diſtended they readily receive the impreſſion of the fingers. The face, arms, and other parts of the body are hydropically ſwollen alſo; but the abdomen does not appear to be aſcitic, or if it contain any fluid, it muſt be in ſmall quantity. The reſpiration is ſo extremely ſhort and laborious as to have diſabled her from lying down for ſome weeks; which, with the other ſymptoms, give every reaſon to ſuppoſe the lungs, if not alſo the cavity of the cheſt, are fully diſtended with ſerous accumulation. She for ſeveral days has been able to reſpire only while the trunk was in an erect poſition, which has confined her to ſitting upon a chair both night and day, in a ſtate of painful and ſuffering exiſtence. Her debility is ſo great that ſhe has hardly the power of ſpeaking to be heard, nor without much attention can it be aſcertained what ſhe articulates. She gets very little ſleep, though from the torpor of the ſyſtem, and the mode of treatment that has been adopted by the adminiſtration of inebriating *stimuli*,* ſhe is, in general, in a ſtate nearly approaching

* Whether the practice alluded to was entirely conſonant to the *Brunonian* doctrines I do not enquire; but that it was in imitation of thoſe doctrines, the following anecdote will demonſtrate; and at the ſame time ſufficiently expoſe the principles of ſuch practice. A phyſician, *(non Salopiæ)* whoſe name is withheld *only* to avoid *controversy*, had been for ſome weeks conſulted ing to comatofe. She has but little if any thirft, and the tongue is moift; fhe paffes fcarcely any urine. Of ftools it was obferved that fhe had generally three or four very fmall ones every day. The pulfe is extremely weak, and cannot be very accurately counted; but I noted the pulfations to exceed 100 in a minute. She complained of having had a ftitch or pain in the left fide of the thorax, in the courfe of this illnefs; and I underftood that, with a little coughing, fhe had expectorated fome bloody mucus, but not in much quantity.

The imminent danger of this patient, reduced by her fymptoms almoft to a dying ftate, requiring fome mode of treatment that would be *fpeedy* and of *certain effect*, I advifed the immediate adoption of fcarifications of the extremities, which were accordingly done at the inftant by a lancet, (not having any more convenient inftrument at hand) fix or eight fmall incifions being made with the point of it on the inner fides near to the ancles of each leg; from which there immediately and copioufly iffued a very kind *stillicidium* of fluid. Warm cabbage leaves were applied over the incifions; fhe took twenty-five drops of *tinctura digitalis*, with direction to repeat them twice a day. For diet fhe was directed beef tea, fago, and winenegus; fhe was not capable of fwallowing any folid.

11th. As fhe lived feveral miles diftant from me, profeffional engagements

---

fulted by this patient before I faw her. He had ordered her to drink *plentifully* of *ftrong old beer*, of which a cafk that had been brewed three years, and was of a ftrength little inferior to brandy, was actually tapped for her ufe exclufively; and left this good old beverage fhould not be fufficiently potent, fhe was alfo directed to ingurgitate freely, every night, of ftrong *Holland's gin* and water, both which injunctions had been carefully followed for a long time. And that her former furgeon, a gentleman of judgment and refpectability in his profeffion, who had attended her in this illnefs, might not be benefited by the Doctor's confultation or prefcription, the family were directed themfelves, at confiderable labour, and fome expence, to collect broom *(genifta)* for burning; the afhes of which were recommended by this phyfician as a fovereign remedy for the difeafe. But when I told the hufband of the patient, who enquired of me my opinion of the remedy, that, inftead of employing his fervants nearly a day in this fiery procefs, he might for a few pence have obtained the fame medicine, and in a much cleaner, purer ftate, in common *pearl afhes*, I know not which moft to admire, the aftonifhment of my non-chemical friend, or the illiberal conduct of the phyfician. But, left the excitement of the fyftem fhould not be entirely complete by *old beer*, and *gin*, and *afhes*, the *diffufible ftimuli* were reforted to, which of courfe led to a liberal employment of *æther*, *volatile alkali*, &c. And that the effufed fluid might be repelled, or expelled, as well by external as internal means, the extremities, *when fwollen as full as the fkin could contain*, were directed to be firmly and conftantly delegated with a flannel roller; the injurious confequence of which foon appeared, and need not be pointed out to the difcerning practitioner. W. C.

engagements prevented my feeing her yefterday. I found this morning that fhe had taken four dofes of the tincture, but without its producing any increafe of urine; yet the pulfe was reduced to only 60 pulfations in the minute. The difcharge from the incifions was going on well, and it was fuppofed that fome *quarts* had drained off, and with the beft effect, for the fwellings of the thighs were much reduced, and the breathing evidently better. She continues to have three or four fmall ftools daily. Directed the drops to be continued, and an analeptic diet.

12th. She was taken very ill this morning about 3 o'clock, (now twelve hours fince) with extreme ficknefs and vomiting. From that time till the prefent fhe has been in a conftant ftate of languor and faintnefs, with extreme debility, coldnefs of the fkin, and increafe of dyfpnœa; the pulfe fo fmall and quick as not to be counted, though fome weak re-action of the arterial fyftem had now taken place. She paffes fcarcely any urine, but the fcarifications continue draining. After waiting two hours, in which time cordials, &c. were adminiftered, the pulfe became more diftinct, and could barely be difcovered, beating about 108 in a minute. Directed only cordials to be given at prefent.

13th. Has had fome increafe of urine to-night; expectoration tinged with blood; the pulfe is lefs quick, and the natural heat of the furface is reftored; in other refpects the fymptoms are nearly ftationary. Complains of pain and weaknefs in the loins. The difcharge of one of the legs having almoft ceafed, the fcarification was repeated; and *bals. Canad.* was fubftituted for the Digitalis, of which fifteen grains were directed to be taken three times a day; and a liniment for the loins, confifting of camphorated fpirit and tincture of cantharides, to be applied twice a day.

14th. Scarification of the other leg was this day repeated, as the former incifions were healed.

18th. She has continued on the whole fomewhat better fince the 14th; has refpired with lefs difficulty, and been able to lay down a little in the night; has had lefs of languor, and has taken more food. Confiderable difcharge going on from the fcarifications; and the fwellings of the legs and thighs are diminifhing.

19th. She had feveral cold chills yefterday evening. The breathing this morning is very difficult, with a ftitch in the left fide of the thorax; p. 132; complains of thirft; has fome increafe of the fwellings of the legs, and makes lefs urine; is alfo purged. A bliftering plafter was applied to the fide, and a draught, confifting of 30 grains of acetated kali, 20 drops of tincture of fox-glove, 4 drops of tincture of opium, and 40 drops of fpirit of nitrous æther, directed to be taken twice a day.

21ft. She has taken four draughts. The dyfpnœa is much better; has not fpat any blood thefe two days till this morning, when fhe expectorated one large clot; coughs now and then, but expectorates without much difficulty; p. now only 102 to 108; has paffed a pint of urine this morning; pain of the fide fhifted higher; fleeps much; ftools regular; the fwellings again leffening. Ordered the draught to be repeated every night only, and a mixture of camphire, vitriolic æther, and fquill, to be taken occafionally to relieve the dyfpnœa.

23d. Continues to mend every day, and has had tolerable good nights for fome time, being enabled to lay down all the night; ftrength and appetite improving; is this day menftruating; urine excreted in proper quantity. The legs are now fo little fwelled as only to require rolling, which they bear well; the cuticle is peeling off them.

25th. She is now able to walk into another room, and is fo far convalefcent as to indicate the propriety of a tonic plan of treatment, which commenced this day. The diuretic draughts were alfo continued every night for nearly two weeks longer, with a fpirituous embrocation occafionally to the legs, previoufly to applying the roller.

On the 20th of May, I had the pleafure to leave her in perfect health, in every refpect except debility and lofs of flefh, both of which fhe was then recruiting very faft; and the exercife on horfeback, which fhe had taken fome days before this, a continuance of which I recommended, left no room to doubt of her entire recovery in a fhort time. In the month of July fhe had no dropfical fymptom whatever, and had fo completely regained both her flefh and ftrength, as to be able to fuperintend the affairs of a large family, in which much exercife and exertion were requifite.

*Introductory Observations to the Second Part of the Case.*

When cafes of rare occurrence become the objects of practice with profeffional men, it furely behoves them to lay the facts before the community from time to time, that thus the profeffion primarily, and the public ultimately, may each be benefited thereby. With this view it is that the following ftatement is communicated, to record an additional inftance of an hydropic tumour of the vagina, which in fome cafes of the afcites, appears to be produced by the latter difeafe; and it probably never occurs unconnected with the afcites. I fufpect, however, that fuch combination may have exifted, though perhaps haps rarely, in cafes of afcites in the female fubject, where, from the delicacy of the patient, it may not have been mentioned, and confequently been neither fufpected nor its prefence known.

This fufpicion is founded on an occurrence that lately happened in my own practice: A female patient, of low circumftances but of good moral character, aged only feventeen or eighteen, unmarried, was in the laft ftage of a phthifis; in which fituation fhe became dropfical, chiefly in the abdomen; the afcites increafed, and fhe complained of dyfury. On being *questioned* whether fhe felt any fulnefs or tumour in the vagina, fhe very reluctantly confeffed fhe did. I was permitted to examine it; and immediately difcovered a foft fluctuating tumour, filling the vagina, and beginning to protrude through the external labia. By pufhing two fingers againft it, it receded within the pelvis; but inftantly returned on removing the fingers. The uterus, of a very fmall fize, by introducing the fingers far within the vagina, was diftinctly felt underneath, or on one fide of the tumour; but fhe complained of fo much pain from the examination that I could not be fatisfied of the whole cafe fo correctly as I wifhed; though I was fully convinced of its being a true inftance of the hydrops vaginæ. She was fo reduced, and in other refpects with the phthifis fo nearly exhaufted, that no operation was propofed; and if it had, would not probably have been allowed. She died a few days afterwards. But that fimilar inftances of the hydropic difeafe are not often met with, may reafonably be prefumed from the entire filence of medical writers on the fubject until very lately; for no account of the fact was ever publifhed in this country, fo far as I know of, until a paper on the fubject by Mr. Watfon, appeared in the firft Volume of "Med. Communications." He defcribes the difeafe as he obferved it in two patients; one of whom he tapped in the vagina twice, the other once, with the effect of curing the afcites in both of them. His directions refpecting the operation are full and judicious. In the fecond volume of the fame work is communicated a fimilar cafe by Sir W. Bifhop, which was treated in the manner recommended by Mr. Watfon, and with equal fuccefs. Dr. Denman, in his Lectures, ufed to defcribe this fwelling as a dropfy of the *perinæum*. He has fince publifhed his fentiments on the fubject; and the theory of it he has advanced, is, without doubt, the only proper explanation the cafe will admit of; which is, that the tumour or prolapfus is "occafioned by the preffure of the water contained in the cavity of the abdomen upon the inflected part of the peritonæum, between the vagina and rectum." (*Introd. to Midwifery*,

 *Vol.*

*Vol. I. p.* 141) He met with an example of this tumour in a pregnant woman; on account of which incident the operation was deferred until after the delivery, and ultimately until after her death, as ſhe died on the fourth day of her lying-in. After this happened, a trocar was puſhed into the tumour, and thus the nature of the diſeaſe was clearly aſcertained; which, until this period, from the profeſſional conduct obſerved, it probably was not; though ſeveral gentlemen of the firſt eminence in the profeſſion were conſulted on the occaſion.

From all theſe circumſtances, it is apparent that it ſhould always be a part of the Surgeon's inquiry, in the examination of female patients afflicted with aſcites, to aſcertain whether at the ſame time there be preſent any tumour or fulneſs of the vagina of the hydropic kind, as ſuch accumulation of fluid would moſt likely be not a mere local tumour, but a continuation of the diſeaſe of the abdomen; requiring of courſe a treatment different to what would be proper for the aſcites only. The operation of tapping the vagina, while it effectually diſcharges the whole fluid of the abdomen as well as that of the vaginal tumour, is ſo much more *safe* and *certain* than the *paracentesis abdominis*, that it ought always to be preferred where the diſeaſe exiſts in this complicated form. For the latter operation, though one of the moſt ſimple in ſurgery, proves ſometimes alſo one of the moſt fatal; and there does not ſeem to be any care or attention on the part of the operator that can prevent it; of which ſee ſeveral inſtances adduced by Mr. Ford, in "Medical Communications, Vol. II." The ſame alſo has been obſerved by Mr. Cline in his Lectures on Surgery.

*Part Second, which comprises an Account of the Ascites and the Hydrops Vaginæ.*

1800, Nov. 3, I was deſired to ſee the above patient again, in conſequence of ſome troubleſome ſymptoms, which ſhe and her female friends ſuppoſed to be dependent upon pregnancy. I found the principal circumſtance ſhe had to mention was a painful tumour in the vagina, which ſhe conceived to be a bearing down of a child's head. She deſcribed it as being moſt troubleſome in the night, and that ſhe found it increaſing in bulk for more than a month, accompanied with frequent difficulty in making water. The abdomen had gradually increaſed in ſize for at leaſt three months, and was now as large as it had uſually been in her former pregnancies. She was very well in all other reſpects. In examining the abdomen, which was fully and equally diſtended, I had no heſitation in believing that the caſe was an *ascites*; and told her ſo. The tumour

I found

I found entirely filling up the vagina, and it was defcended full three inches below its external orifice. On preffing againft it with the fingers it readily receded; and it indubitably contained a fluid. This tumour was a part of the vagina, the pofterior portion of it forming an evident *prolapsus vaginæ*; which was afcertained by paffing the fingers over it, from the perinæum upwards. As the whole of the vagina was thus filled, it evidently occafioned compreffure upon the urethra, thereby occafioning the dyfury of which fhe complained. By pufhing the prolapfus upwards, the contained fluid readily paffed into the pelvis; but on removing the hand it inftantly returned. I had no doubt but this fwelling was occafioned by the weight or preffure of the fuperincumbent water of the afcites; and therefore, in fact, that only one difeafe exifted, though two feparate, but not unconnected hydropic tumours were formed; which circumftances I mentioned at the time, and encouraged her to hope for a perfect cure of all the fymptoms by an operation. To this fhe readily confented, being defirous to fubmit to any treatment that would be likely to relieve her from the pain and trouble fhe had fo long endured. The uterus was not within reach of the fingers.

Nov. 5. Previously to the perforation, which was performed this day, I made a more full and ocular examination of the difeafe; from which it was demonftrably afcertained that the tumour was formed of the pofterior part of the vagina. The external fubftance was flefhy, and the cuticle had peeled off it fome time before, as it had now a furfuraceous appearance remaining. The operation was performed in the manner recommended and defcribed by Mr. Watfon. (*Med. Commun. Vol. I. Art.* 12.) A fmall round trocar was ufed; and I found it neceffary before paffing it, to introduce two fingers of the left hand over the tumour to prevent the retro-ceffion of the fluid and make the fubftance more tenfe. This precaution feems highly requifite. She ftood upright during the operation, leaning her back againft the back of a chair, fupported by two affiftants. The fluid, which was of a deep ftraw colour and inodorous, immediately paffed through the canula in a full ftream. After about three quarts had difcharged, an accident difplaced the canula from the wound; but without any kind of inconvenience, as the difcharge continued equally well to the very laft, excepting occafional interruptions that occurred from her fitting down, which fhe was allowed to do once or twice. Changing the upright pofture, we found immediately prevented the liquid flowing out; notwithftanding which, the whole quantity, amounting to two gallons, was thus evacuated in about half an hour. Preffure was applied to the abdomen during during the whole time, both to prevent fainting and to render the evacuation as complete as could be. Afterwards a dry flannel roller was applied to the belly, and a warm cloth to the pudenda. No dreffing or application of any kind was made ufe of; and fcarcely a drop of blood was effufed from the wound. After the operation I perceived a fmall circumfcribed tumour remaining in the hypogaftrium, which I fufpected to be an hydatid; but except this there was a very complete collapfe both of the abdomen and of the tumour. She bore the evacuation very well and was afterwards put to bed.

On the next day I found fhe had paffed an indifferent night, having had feveral fpafmodic pains in the belly; but they were now gone off. She had made water twice with perfect eafe; and had not felt any pain or inconvenience in the punctured part. She was ordered a cordial diet, and the abdomen to be rubbed every morning with camphorated fpirit, continuing the bandage; alfo a diuretic medicine. She got up this morning to breakfaft.

7th. She thinks the fwelling of the hypogaftrium fomewhat increafed; no other alteration.

12th. She has taken only three dofes of the medicine, (*pul. digit.*) having intermitted it on account of its occafioning naufea. The fwelling in the vagina is beginning to return, which fhe firft perceived laft night; and fhe thinks the abdomen is again filling. Health good.

19th. Since fhe has taken the medicine regularly, which I defired fhe would do, the fwelling of the abdomen has diminifhed; and fhe feels lefs of the tumour in the vagina.

26th. Completely cured and in good health. The medicine produced a diuretic effect, and confequently occafioned abforption of the remaining part of the effufed fluid.

She continued perfectly well for more than five months after the operation; but fo ftrongly rooted was the hydropic diathefis in her conftitution, that after this time the dropfical fymptoms returned in their original form; and fhe again became affected with dyfpnœa and œdema of the extremities. Thefe were feveral times relieved or removed by internal remedies, and occafionally by fcarifications; but fhe feldom kept tolerably well many weeks together without medical help. In the month of November laft all the fymptoms became worfe; and under this affliction, with every affiftance that art could fuggeft, fhe found no relief. After thus lingering fome weeks fhe at length died, perfectly exhaufted and worn out; but fhe never had any return of the afcites, or of the hydrops vaginæ. No diuretic ever gave her fo much relief in various ftages of her illnefs as the digitalis; and the effect of this was fometimes increafed by combining combining it with hydrargyrus; it however at length, from frequent repetition, became quite uſeleſs, or acted only as an emetic.

*Bridgnorth, Feb.* 1802. W. C.

